# Pregnancy‐related knowledge in women with epilepsy in childbearing age: A pilot questionnaire survey from China

**DOI:** 10.1002/brb3.3400

**Published:** 2024-02-10

**Authors:** Qiwei Li, Yina Cao, Jingxin Zhang, Yanlu Fu, Beibei Shen, Shuang Wang, Jiajia Fang

**Affiliations:** ^1^ Department of Neurology, The Fourth Affiliated Hospital, International Institutes of Medicine Zhejiang University School of Medicine Yiwu P. R. China; ^2^ Department of Neurology, The Second Affiliated Hospital Zhejiang University School of Medicine Hangzhou Zhejiang P. R. China

**Keywords:** antiseizure medications, knowledge, pregnancy, questionnaire, women with epilepsy

## Abstract

**Purpose:**

We aim to understand the knowledge of and attitudes toward pregnancy issues among women with epilepsy (WWE) and their caregivers and analyze the answers from the questionnaire to expose topics that require educational activities; thus, WWE experiences pregnancy better.

**Methods:**

WWE at their childbearing age and/or their caregivers who entered the Fourth Affiliated Hospital of Zhejiang University for treatment of their condition were invited to fill out a questionnaire between March 1 and November 31, 2022.

**Results:**

A combined total of 205 WWE and 142 caregivers completed the questionnaires. Among the surveyed WWE, a majority (63.74%) reported experiencing at least one miscarriage or induced abortion. However, a significant proportion (84.62%) of these WWE were still able to successfully give birth to at least one child. Furthermore, the offspring of these WWE showed no significant differences compared to the offspring of women without epilepsy, as reported by 93.51% of the participants. The participants’ knowledge regarding the impact of epilepsy on pregnancy was found to be comparable, with average scores of 7.74 and 7.84, respectively. The participants exhibited a limited comprehension of antiseizure medications (ASMs)‐related knowledge, specifically pertaining to ASMs adjustment during pregnancy (17.56% vs. 16.90%) and offspring outcomes (30.24% vs. 26.06%). Statistical analysis revealed significant correlations between the overall score and education level (*p* < .001), as well as epilepsy duration (*p* = .008). Regarding the source of knowledge, participants acknowledged primarily relying on neurologists, who remained their preferred choice for consultation.

**Conclusion:**

In our study, the understanding of pregnancy‐related knowledge did not differ from WWE and their caregivers, both are far from satisfactory in certain areas. It is urgent for WWE and their caregivers to improve their pregnancy‐related knowledge of epilepsy. As their primary access is from knowledgeable health care professionals like neurologists, well‐trained neurologists in epilepsy management during pregnancy are in need.

## Graphical abstract

Women with epilepsy who had a higher education level and longer epilepsy duration obtained higher scores on the questionnaire. This phenomenon suggests that participants primarily acquired pregnancy‐related knowledge of epilepsy through their own initiative rather than through guidance from healthcare professionals. Our study provides a valuable point of reference for the future development of health literacy in China.

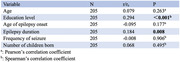

## 1 LIMITATIONS

Several limitations also exist in our study: First, the participants were recruited exclusively from a single‐tertiary hospital, which may limit the generalizability of our findings. Second, the presence of selection bias is evident as some of patients and caregivers declined to participate in the study, potentially influencing the representativeness of our sample. Additionally, in order to mitigate a high dropout rate, we exclude certain detailed questions from the questionnaire. Consequently, we are unable to examine the potential relationship among knowledge, detailed epilepsy history, and the dosage of ASMs. Third, the reproducibility of the questionnaire was not assessed among the surveyed group.

## 2 INTRODUCTION

Epilepsy is a prevalent chronic neurological disorder globally, with an estimated lifetime prevalence of 6.85 cases per 1000 women (Fiest et al., 2016). A significant proportion of women affected by epilepsy are of childbearing age and thus encounter gender‐specific challenges, particularly during pregnancy. Although the majority of women with epilepsy (WWE) experience uneventful pregnancies, deliveries, and successful childbirth, research has demonstrated an increased risk of pregnancy complications and adverse offspring outcomes (Stephen et al., 2019). Notably, studies have revealed that over 1.3 million WWE in the United States are currently in their reproductive years (Li et al., 2021). Despite the limited availability of Chinese demographic data, the implications of managing epilepsy during pregnancy for WWE, their families, medical practitioners, and society at large are significant, particularly in light of China's vast population and the gradual implementation of the two‐ and three‐child policy.

In China, it is customary for unmarried women to reside with their parents. Chinese parents possess a longstanding tradition of providing care for their offspring, irrespective of their age, particularly as WWE are in a disadvantaged position. Consequently, the perceptions of WWE may be partially shaped by the influence of their parents. Upon entering into matrimony, WWE and their partners assume joint responsibility for procreation. Given that epilepsy can potentially impair cognitive and social abilities of WWE, spouses are required to assume a heightened level of responsibility, including acquiring a comprehensive understanding of pregnancy‐related cognition.

Given these complex considerations, preconceptual counseling plays a pivotal role in the management of WWE, especially regarding effective birth control, planned pregnancies, and antiseizure medications (ASMs) optimization. However, a study in the USA has shown that half of the pregnancies in WWE are unplanned (Johnson et al., 2018). Thus, it is essential to know the conception of pregnancy‐related knowledge in WWE and their caregivers and to take regular preconceptual counseling.

Accordingly, the aims of this study were as follows: (1) conduct a questionnaire survey to understand if WWE and their caregivers have comparable knowledge of and attitudes toward pregnancy issues, (2) try to analyze the answers from the questionnaire to expose topics that require future educational activities.

## 3 MATERIALS AND METHODS

This is a single‐center study conducted in the Fourth Affiliated Hospital, Zhejiang University School of Medicine from March 1 to November 31, 2022. The ethics committees of The Fourth Affiliated Hospital, Zhejiang University School of Medicine approved all the procedures (K2022055). The participants provided informed consent after being briefed on the study's objectives and guaranteed confidentiality. Participants were also granted the prerogative to dissent from participation and discontinue their involvement at any given point.

### 3.1 Participants and definitions

All consecutive WWE and/or their caregivers who entered the epilepsy of the outpatient or inpatient department and met the inclusion criteria were subjected to in‐person interviews. Caregivers were defined as family caregivers who accompany WWE to the clinic and live together for at least 3 months after their patients were diagnosed with “epilepsy.” Participants are required to enter a designated room and subsequently scan a QR code to complete an electronic questionnaire. After completing the questionnaire, a self‐written brochure about pregnancy‐related knowledge was distributed to them with personalized education guidance and the general feedback was positive. The inclusion criteria were as follows: (1) were at their childbearing age, which we set the age at 18–49 years in our study according to Chinese social demography; (2) were diagnosed with epilepsy for more than 1 year which met the diagnostic criteria of revised 2014 definition of the International League Against Epilepsy (Fisher et al., 2014); (3) informed consent obtained. Patients were excluded if they had a history of mental retardation or uncontrolled psychosis, or if they were unable to understand the questionnaires. For those caregivers with informed consent who were asked to fulfill the questionnaire, they should be (1) at least 18 years old; (2) understand the questionnaires; (3) the WWE with whom they accompany should meet the aforementioned inclusion criteria at the same time.

### 3.2 Survey instruments

#### 3.2.1 Questionnaire on demographics and clinical data

Part one of the questionnaire was custom‐designed for this study to collect information. The questionnaire that was to be administrated to WWE comprised three sections: (1) demographic data (age, education level); (2) personal information about pregnancy (e.g., contraceptive methods, history of pregnancy, miscarriage or abortion, and delivery); (3) history of epilepsy (e.g., age of epilepsy onset; present frequency of seizures; type of seizure; present ASMs prescribed). As patients had to fulfill their history of epilepsy with/without their caregivers’ help, the type of epilepsy was simply classified as with impaired awareness or not. For those patients with both types of seizure, they were asked to choose the option of with impaired awareness if they lost consciousness in the last 12 months.

The other questionnaire, which was to be administrated to caregivers, only comprised two questions about their identity and education level to increase the response rate of the questionnaire.

#### 3.2.2 Questionnaire on pregnancy‐related knowledge

Part two of the questionnaires was largely analogous in both two groups, which consisted of 13 single‐choice questions and three multiple‐choice questions about pregnancy‐related knowledge. Each single‐choice question was designed to offer three or four potential response options, of which only one was considered accurate based on current medical knowledge, with the exception of question 7. As patients were directed to keep seizure‐free for 12 months at least before pregnancy in practice in China while studies figured out that keeping seizure‐free for at least 9 months is ideal before pregnancy (Metcalfe et al., 2012), we set both options as a correct answer. Those 13 single‐choice questions are scored on the basis of the principle of awarding one point for correct responses and no points for incorrect ones. Patients were instructed to indicate the options they deemed most suitable. Additionally, the final three multiple‐choice questions were open‐ended and not included in the scoring process.

### 3.3 Statistical analysis

The statistical analysis was conducted using SPSS version 26.0 (Statistical Package for Social Sciences, IBM). The mean ± standard deviation was used to report quantitative data, whereas percentages were employed to present categorical variables. The comparison of results was performed using either the chi‐square test or Fisher's exact test for categorical variables, and Student's *t*‐test for continuous variables to assess differences between groups. Chi‐squared tests were employed to ascertain the associations between nominal variables to compare the knowledge levels between WWE and their caregivers. To examine the disparities in means, independent samples *t*‐tests, Mann–Whitney *U* tests, or one‐way analysis of variance were conducted. Pearson's r and Spearman's rho were computed to assess the relationships between variables. All *p* values were two‐tailed, with a significance level of *p* < .05.

## 4 RESULT

### 4.1 Demographic and clinical data

#### 4.1.1 Group demographics

After a 9‐month investigation, a total of 205 WWE (response rate 93.2%) and 142 caregivers (response rate 93.4%) successfully completed the questionnaires. The comprehensive demographic information is presented in Table 1. A significant proportion of WWE (*n* = 99, 48.29%) reported possessing a college education or higher, whereas the majority of caregivers (*n* = 48, 33.80%) disclosed having completed only junior high school education (*p* < .001).

**TABLE 1:** Sociodemographic characteristics of participants.

Characteristics	Women with epilepsy			Caregivers			*p*‐Value
**Age**	*N*	Mean	SD				
	205	29.4	7.1				
**Role**				142	*N*	%	
Spouse					68	47.89	
Parents					54	38.03	
Others					20	14.08	
**Education level**	205	*N*	%	112	*N*	%	**<.001**
≤6 years		6	2.93		17	11.97	
6–9 years		50	24.39		48	33.80	
9–12 years		50	26.39		33	23.24	
>12 years		99	48.29		44	30.99	
Score of questionnaire^a^	N	Mean	SD	N	Mean	SD	
	205	7.79	2.73	142	7.80	2.39	.971

^a^ Calculate the scores for question1–13 in the questionnaire.

In the surveyed WWE, it was found that 21.96% did not utilize any form of contraception, whereas 3.41% relied on oral contraceptives. The WWE group was evenly divided between those with a history of pregnancy and those without (50.24% vs. 49.76%). In the former group, a significant proportion (63.74%) reported experiencing at least one instance of miscarriage or induced abortion. However, the majority (84.62%) still successfully gave birth to one or more children, and only 6.49% of the offspring displayed abnormalities in their reproductive development or intelligence. Detailed data are presented in Table 2.

**TABLE 2:** Clinicodemographic characteristics of participants.

Characteristics	Women with epilepsy	
Contraceptive method (*N* = 205)	*N*	%
No contraception	45	21.96
Condoms	89	43.41
Oral contraception	7	3.41
Others	36	17.56
No sex	28	13.65
**History of pregnancy (*N* = 205)**	***N***	%
No	102	49.76
Yes	103	50.24
**Number of miscarriages or abortion (*N* = 103)**	***N***	%
0	36	36.26
1	46	39.56
≥2	21	24.18
**Number of children born (*N* = 103)**	***N***	%
0	14	15.38
1	53	50.55
≥2	36	34.07
**Abnormal offspring (*N* = 89)**	***N***	%
No	84	94.38
Yes	5	5.62
**Age of epilepsy onset**	Mean	SD
	19.0	9.5
**Epilepsy duration**	Mean	SD
	10.4	7.9
**Frequency of epilepsy (*N* = 205)**	***N***	%
Several times per month	54	26.34
Several times per year	75	36.59
Seizure free for almost 1 year	31	15.12
Seizure free for several years	45	21.95
**Seizure type (*N* = 205)**	***N***	%
With impaired awareness	170	82.93
Without impaired awareness	35	17.07
**Current on ASM treatment (*N* = 205)**	***N***	%
No	19	9.27
Yes	186	90.73
**ASM treatment (*N* = 186)**	***N***	%
Monotherapy	95	51.08
Polytherapy	91	48.92
**ASM treatment (*N* = 186)**	***N***	%
Carbamazepine	24	27.91
Oxcarbazepine	64	34.41
Levetiracetam	82	44.09
Lamotrigine	66	35.48
Valproic acid	27	14.52
Topiramate	14	7.53
Lacosamide	16	13.98
Perampanel	19	10.22
Other	7	3.76

Abbreviation: ASMs, antiseizure medications.

The mean epilepsy duration was 10.4 years (standard deviation 7.9 years, range 1 to 33 years). The majority of patients (62.93%) continue to experience epilepsy at least once per year, 82.93% (*n* = 170) of patients reported experiencing seizures with impaired awareness, 90.73% (*n* = 186) continue to adhere to regular ASMs usage, and 51.08% (*n* = 95) of WWE were subject to monotherapy. The commonly prescribed ASMs include levetiracetam (44.09%), lamotrigine (35.48%), and oxcarbazepine (34.41%). Notably, 14.52% (*n* = 27) are still undergoing treatment with valproic acid (VPA). Detailed clinicodemographic characteristics of these 27 WWE are listed in Table S1.

### 4.2 Pregnancy‐related knowledge

The median scores of questionnaires for WWE and caregivers were 7.74 and 7.84, respectively, as shown in Table 3. Among these two surveyed groups, the highest percentage of correct answers was observed for questions 5 (informed obstetrician of a history of epilepsy: 97.56% vs. 95.07%) and 4 (prenatal consultation: 92.20% vs. 96.48%). However, when examined for a more detailed understanding of the impacts, they demonstrated poor performance (16%–78% accuracy). In both groups, the issues of ASM adjustment during pregnancy, offspring birth outcomes, pregnancy complications, and breastfeeding are the most problematic.

**TABLE 3:** Questionnaire of pregnancy‐related knowledge.

Question	Patients		Caregivers		*p*‐Value
	*N*	%	*N*	%	
**1. In your opinion, is epilepsy always a hereditary disease?**					.498
Yes	14	6.83	14	9.86	
**No**	139	67.80	97	69.31	
I don't know	52	25.37	31	21.83	
**2. In your opinion, can women with epilepsy be pregnant?**					.599
**Yes**	145	70.73	104	73.24	
No	17	8.29	14	9.86	
I don't know	43	20.98	24	16.90	
**3. If women with epilepsy can be pregnant, in your opinion, is planned pregnancy important?** ^a^					.070
**Important and necessary**	114	78.62	70	67.31	
Important but not necessary	24	16.55	22	21.15	
Unimportant	7	4.93	12	11.54	
**4. If you/your relative plan to be pregnant, will you consult your doctor?**					**.012**
**Yes**	189	92.20	137	96.48	
No	3	1.46	4	2.82	
I don't know	13	6.34	1	0.70	
**5. In your opinion, is it necessary to inform your obstetrician of your (relative's) history of epilepsy?**					.503
**Yes**	200	97.56	135	95.07	
No	2	0.98	2	1.41	
I don't know	3	1.46	5	3.52	
**6. In your opinion, is it necessary to supplement with folic acid from the beginning of pregnancy preparation for women with epilepsy?**					.889
**Yes**	135	65.85	94	66.20	
No	3	1.46	3	2.11	
I don't know	67	32.68	45	31.69	
**7. In your opinion, how long at least should women with epilepsy remain seizure‐free before conception?**					.264
6 months	25	12.20	13	9.15	
**9 months**	4	1.95	4	2.82	
**12 months** ^b^	91	44.39	77	54.23	
I don't know	85	41.46	48	33.80	
**8. In your opinion, should women with epilepsy keep taking antiseizure medications during pregnancy?**					.492
**Yes, and even increase the dose if necessary**	36	17.56	24	16.90	
Yes, but decrease the dose	77	37.56	43	30.28	
No	36	17.56	30	21.13	
I don't know	56	27.32	45	31.69	
**9. In your opinion, should women with epilepsy monitor the concentrations of anti‐seizure medications during pregnancy?**					.391
**Yes**	160	78.05	103	72.54	
No	2	0.98	3	2.11	
I don't know	43	20.98	36	25.35	
**10. In your opinion, do women with epilepsy have a higher risk of complications during pregnancy compared to general populations (e.g. bleeding, premature, cesarean birth)?**					.473
**Yes**	107	52.20	67	47.18	
No	21	10.24	20	14.09	
I don't know	77	37.56	55	38.73	
**11. In your opinion, do women with epilepsy have a lower probability of having a healthy baby compared to general populations?**					.442
**Yes**	62	30.24	37	26.06	
No	73	35.61	60	42.25	
I don't know	70	34.15	45	31.69	
**12. In your opinion, can women with epilepsy have a natural childbirth?**					.878
**Yes**	113	55.12	82	57.75	
No	28	13.66	19	13.38	
I don't know	64	31.22	41	28.87	
**13. In your opinion, can women with epilepsy breastfeed babies in most cases?**					.326
**Yes**	92	44.88	75	52.82	
No	52	25.37	29	20.42	
I don't know	61	29.76	38	26.76	
**14. In your opinion, what is the greatest impact on the growth and development of the fetus? (Select one or two top options)**					
Antiseizure medications	121	59.02	83	58.45	
Seizure during pregnancy	101	49.26	61	42.96	
Heredity	76	37.07	51	35.91	
Pregnancy complications	48	23.41	31	21.83	
**15. What is your source of information about pregnancy‐related knowledge? (multi‐choice)**					
Consult health care professionals	185	90.24	130	91.55	
Information online	59	28.78	57	40.14	
Wardmate	23	11.22	21	14.79	
Other	34	16.59	18	12.68	
**16. Where do you want to get more information about pregnancy‐related knowledge in the future? (multi‐choice)**					
Health care professionals	193	94.15	137	96.48	
Related professional books or promotional materials	109	53.17	68	47.89	
Wardmate or others	40	19.51	26	18.31	
No need	6	2.93	4	2.82	

^a^ People who select “No” or “I don't Know in question 3” will skip this question.

^b^ We also make “12 months” as correct answer as patients were directed to keep seizure‐free for 12 months at least before pregnancy in practice.

Besides, an evaluation was conducted to determine if the accuracy of the 13 questions was influenced by prior pregnancy experience. The findings revealed that a higher percentage of women with a history of pregnancy correctly answered question 6 regarding folic acid supplementation (76.70% vs. 54.90%, *p* < .001). Conversely, a greater proportion of women without pregnancy experience correctly answered question 13 regarding breastfeeding (40.78% vs. 49.02%, *p* < .001). No significant association was observed between two groups in the remaining 11 questions.

Furthermore, our research investigates the influence of sociodemographic variables, disease advancement, and social role on the questionnaire score. Moreover, we analyze the correlation between score and sociodemographic factors, along with the frequency of seizure types, as presented in Tables 4 and 5, correspondingly. Only some certain weak but statistically significant relationships existed: positive with educational level (*p* < .001) and epilepsy duration (*p* = .008).

**TABLE 4:** Mean values of correct answers about seizure control, type of epilepsy, pregnancy experience, antiseizure medication (ASM) therapy, and social role.

		*N*	Mean	SD	*p*‐Value
Seizure control	>1 seizure/year	129	7.62	2.62	.419^a^
	No seizure in the last 12 months	76	7.95	2.89	
Type of epilepsy	With awareness impaired	170	7.78	2.72	.685^a^
	Without awareness impaired	35	7.57	2.77	
Pregnancy experience	No pregnancy	103	7.90	2.32	.394^a^
	Pregnancy	102	7.58	3.08	
Number of children born	0	14	7.64	2.47	.890^b^
	1	53	7.98	2.06	
	≥2	36	7.89	2.65	
ASM therapy	Monotherapy	95	7.62	2.75	.498^b^
	Polytherapy	91	7.97	2.68	
	No ASM	19	7.26	2.79	
Social role	Spouse	68	8.03	2.04	.415^b^
	Parents	54	7.70	2.46	
	Others	20	7.25	2.69	

^a^ Independent sample *t*‐test.

^b^ One‐way ANOVA.

**TABLE 5:** Correlation of pregnancy‐related knowledge with demographic factors, history of epilepsy, and history of pregnancy.

	*N*	*r*/*r*_s_	*p*‐Value
Age	205	.079	.263^a^
Education level	205	.294	**<.001** ^b^
Age of epilepsy onset	205	−.095	.177^a^
Epilepsy duration	205	.184	**.008**
Frequency of seizure	205	−.008	.906^b^
Number of children born	205	.068	.495^b^

^a^ Pearson's correlation coefficient.

^b^ Spearman's correlation coefficient.

## 5 DISCUSSION

We conducted a questionnaire survey among those WWE and/or caregivers who entered our outpatient or inpatient epilepsy clinic to seek medical care. The key findings of our study were as follows: (1) Approximately half of WWE in our study possessed a prior pregnancy record, and the majority achieved successful deliveries. (2) The most commonly prescribed ASMs were levetiracetam, lamotrigine, and oxcarbazepine. Notably, nearly one in six WWE still use VPA. (3) Participants’ pregnancy‐related knowledge of epilepsy was comparable and did not reach the desired level yet, the most problematic questions were ASMs adjustment during pregnancy and offspring outcomes. (4) Most of participants admitted their pregnancy‐related knowledge primarily came from neurologists, and neurologists remained their preferred source for consultation. (5) Education level and epilepsy duration had an independent association with the score of questionnaires in WWE.

### 5.1 Fertility status of the surveyed WWE

In our study, 50.24% of WWE had a history of pregnancy. This finding aligns closely with the reported the prevalence of pregnancy history among women of childbearing age in China, which reported a rate of 54.1% (Institute of Fudan University Health Communication, 2022). It suggests that the fertility rates of WWE in China were comparable to general population, thereby contradicting previous studies (Cheravalloor, 2010; Farmen et al., 2016; MacEachern et al., 2019), which suggested a decline in fertility among WWE. One factor contributing to the elevated fertility rates can be attributed to the failure of our study to differentiate whether epilepsy was present prior to pregnancy. Nevertheless, a recent investigation similarly revealed no discernible disparity in the median duration to conception between WWE and controls (Pennell et al., 2018).

In WWE with a history of pregnancy, it is observed that 84.62% have given birth to one or more children, a percentage lower than that of the general female population in China (99.47% incidence) (Institute of Fudan University Health Communication, 2022). Additionally, our survey findings indicate a 63.74% occurrence of abortions or miscarriages among the surveyed population, which potentially contributes to the observed lower birth rate. In our study, it was observed that approximately 25% of women with a history of pregnancy in the WWE population experienced repeated abortions. This phenomenon was predominantly observed in the older age group, which aligns with the findings of the general China fertility survey (Wang & Jiang, 2022). Previous studies (Mostacci et al., 2018; Viale et al., 2015) have indicated an elevated likelihood of spontaneous miscarriage among WWE; however, our investigation revealed a significantly higher rate of fetal loss in WWE compared to another study that reported an abortion rate of 11.5% (Mostacci et al., 2018). The factors contributing to the elevated rate of abortion in WWE align with those general Chinese women in some certain (Wang & Jiang, 2022).

Nevertheless, the limited utilization of contraceptives could potentially be a contributing factor to the elevated incidence of abortion in WWE. In our study, it was found that among 177 WWE, only 132 individuals were found to utilize effective contraceptives, which was lower than the reported contraceptive usage rate of 79.2% within the general population of China (Haakenstad et al., 2022). However, doctors rarely ask WWE about their contraceptive use in clinical practice. Although the guidelines in the United Kingdom have recommended the provision of appropriate contraceptive counseling to WWE prior to engaging in sexual activity, the comprehensive clinical guidelines in China have not yet been made available to the public. It is imperative to acknowledge that the ongoing development of comprehensive Chinese guidelines pertaining to WWE of reproductive age who possess the capacity for childbearing is of utmost significance.

### 5.2 Perception of pregnancy‐related knowledge among participants

There was a lack of statistically significant disparity observed between WWE and caregivers in terms of their scores and unknown incidence, except for the domain of prenatal consultation, where the latter group performed better. Despite patients having higher levels of education compared to caregivers (*p* < .001), both groups appeared a limited comprehension of pregnancy‐related knowledge.

Both patients and caregivers demonstrated a high level of agreements (>90%) on disease informing obstetricians as well as prenatal consultation. This indicates that both groups comprehended the potential adverse consequences of epilepsy and/or ASMs on their own well‐being and the health of their future offspring. However, upon further inquiry, it became evident that neither of the two groups possessed a comprehensive understanding of the appropriate adjustment pattern for ASMs. In spite of variations in the options available, prior research conducted in Poland and Canada (Jędrzejczak et al., 2020; Metcalfe et al., 2012) has demonstrated that a majority of WWE were aware of the fluctuating clearance of ASMs during pregnancy. These findings highlight the necessity of seeking professional guidance to appropriately adjust ASM dosages. The precise cause of this phenomenon remains uncertain, with potential explanations including the following: (1) A significant disparity in medical accessibility and public health education between developed and developing countries. (2) Underestimated negative consequence of epileptic seizure during pregnancy: generalized tonic–clonic seizures lead to maternal hypoxia, lactic acidosis, and fetal asphyxia during pregnancy (Haakenstad et al., 2022), let alone increased frequency of seizures during pregnancy (Pennell et al., 2020). (3) Overestimated of the negative effect of ASMs on the fetus: Past research had suggested that fetal exposure to ASMs heightened the likelihood of major congenital malformations. However, a recent study has demonstrated that, with appropriate selection of ASMs, there was no significant disparity in fetal loss or major congenital malformations between WWE and the control group. The only exception was the occurrence of fetal loss, which was influenced by the level of ASMs and attributed to ASMs polytherapy (Razaz et al., 2017).

Indeed, the ASMs most commonly prescribed in our study include levetiracetam, lamotrigine, and oxcarbazepine. The teratogenicity of these ASMs was found to be comparable to that of the untreated group (Tomson et al., 2018). It is imperative that their health care providers comprehend the advantages of utilizing the appropriate type and dosage of ASMs to mitigate the risk of epileptic seizures during pregnancy, thereby benefiting both the WWE and their offspring.

However, a significant proportion of the participants exhibited a tendency to underestimate the prevalence of adverse birth outcomes and pregnancy complications. This perspective can potentially be attributed to the inclination to implement misguided notions, such as reducing the dosage of ASMs or completely discontinuing their usage. The occurrence of epileptic seizures during pregnancy is correlated with heightened probabilities of unfavorable pregnancy and perinatal consequences (Razaz et al., 2017). Conversely, the utilization of ASMs during pregnancy is generally not associated with adverse maternal and fetal or neonatal outcomes, with the exception of its potential teratogenic effects.

Previous observational studies have established that the seizure‐free duration prior to pregnancy is a significant determinant in forecasting the likelihood of remaining seizure‐free during pregnancy (Battino et al., 2013; Vajda et al., 2008). However, it is noteworthy that only a limited proportion of patients and caregivers (44.38% and 59.82%, respectively) possessed knowledge regarding the specific duration in our study.

Not only intrauterine exposure but also breastfeeding while receiving ASMs is a significant concern for WWE, leading to a lower percentage of WWE breastfeeding their children compared to the general population (Meador et al., 2014). Previous survey findings indicate that most neurologists in our region are aware of the recommendation to encourage breastfeeding in WWE undergoing ASM monotherapy (Xu et al., 2022). However, our study found that only approximately half of WWE and their caregivers (45.51% and 53.57%, respectively) acknowledged the benefits of breastfeeding outweighing the drawbacks, which was not satisfactory yet.

It is imperative for Chinese neurologists to provide related information to these WWE and their caregivers once WWE reach sexual maturity. Furthermore, it is highly encouraged to foster close collaboration and communication between obstetric professionals in order to promote optimal care for these individuals.

### 5.3 Incomprehensive and de‐normalized treatment in China

Over 90% of patients and their caregivers acknowledged that their knowledge regarding pregnancy‐related matters primarily stemmed from their neurologists, and publication of Chinese guidelines on the management of WWE during periconceptional period highlights the endorsement of vaginal delivery and folic acid supplementation (Group of Electroencephalography and Epilepsy, Branch of Neurology, Chinese Medical Association, 2021). However, the findings revealed that only approximately 50%–60% of the individuals surveyed provided accurate responses, indicating the need for further improvement.

In our study, 27 WWE, all of whom were within the reproductive age range, were observed to be using VPA. Although certain WWE may resort to VPA due to the ineffectiveness of alternative ASMs, a notable percentage of women employ VPA as a consequence of neurologists’ limited awareness regarding its detrimental consequences. This particular ASM is recognized as a teratogen and has been linked to adverse outcomes in offspring (Tomson et al., 2018). It is noteworthy that the Food and Drug Administration has persistently emphasized the necessity of a “black box” warning for this drug.

Furthermore, it is noteworthy that over 90% of individuals express a desire to obtain additional information from their health care professionals. However, it is important to acknowledge that only a minority of doctors consistently offer guidance on pregnancy for WWE, in contrast to the majority who provide information upon patient request (Xu et al., 2022). This observation could potentially indicate suboptimal doctor–patient communication, potentially resulting from a focus on maximizing efficiency. Given the high turnover rate in Chinese clinics, the utilization of an online tool, as previously developed by Zelano's group (Lisovska et al., 2021) would be an ideal approach for WWE to access information at any time and location, thereby enhancing patient education and facilitating improved communication.

### 5.4 Factors related to knowledge

Contrary to previous research (Dierking et al., 2018; Metcalfe et al., 2012), which failed to establish a significant association between prior pregnancy experience and knowledge, our study reveals that individuals with previous pregnancy experience exhibit superior adherence to folic acid supplementation. This outcome may be attributed to their personal experiences. In contrast to the findings of a previous study (Jędrzejczak et al., 2020), which indicated that women with prior pregnancy experience exhibited a more favorable response to the inquiry regarding breastfeeding, our study revealed that women without prior pregnancy experience demonstrated superior performance. This observation may be attributed to their own experiences, misguided and outdated. Consequently, it is crucial to provide WWE and caregivers with up‐to‐date knowledge pertaining to pregnancy‐related matters.

The study has successfully identified the factors that exhibited a statistically significant impact on the score, namely, the education level and epilepsy duration. The previous research (Friedrich et al., 2018; Jędrzejczak et al., 2020) has consistently reported a positive correlation between knowledge and education, which is further supported by our findings. In contrast to prior studies that indicated no association between epilepsy duration and pregnancy‐related knowledge (Metcalfe et al., 2012), our study reveals a weak yet statistically significant relationship between the score and epilepsy duration. This phenomenon can be attributed to the enhanced availability of information, enabling individuals with longer durations of epilepsy to acquire more knowledge about their condition.

This suggests that participants primarily acquired pregnancy‐related knowledge through their own initiative rather than through guidance from health care professionals. Epilepsy is one of the most common neurological diseases worldwide, nearly 80% of patients live in low‐ and middle‐income countries, including China. Although the World Health Organization has emphasized the need to prioritize epilepsy within global public health agendas (World Health Organization, 2019), the availability of comprehensive health care system data in China remains inadequate. Consequently, policymakers face challenges in guaranteeing widespread dissemination of epilepsy‐related knowledge. Our study offers a valuable point of reference for the future development of health literacy in China.

## 6 CONCLUSION

In our study, it was observed that both WWE and caregivers exhibited a lack of satisfactory pregnancy‐related knowledge in certain domains. Although they demonstrated awareness regarding the significance of preconception counseling and disclosing medical history, their limited comprehension of intricate details is concerning, particularly in relation to ASMs’ adaptation during pregnancy. The inflated perception of risk associated with ASMs contributes to the inaccurate understanding of ASMs‐related information, including offspring outcomes, pregnancy complications, and breastfeeding, which are areas of particular concern. Enhancing the knowledge of WWE and their support systems is crucial. Although pregnancy‐related information is readily available through open‐access sources, the primary source of information for WWE is health care professionals, particularly neurologists. Consequently, it is imperative to have well‐trained neurologists specializing in epilepsy management during pregnancy, supported by appropriate written materials.

## AUTHOR CONTRIBUTIONS

**Qiwei Li**: Conceptualization; methodology; writing—review and editing; writing—original draft; validation; formal analysis; visualization; software; investigation; data curation. **Yina Cao**: Data curation; formal analysis; resources; investigation. **Jingxin Zhang**: Data curation; formal analysis; investigation. **Yanlu Fu**: Data curation; formal analysis; investigation. **Beibei Shen**: Data curation; formal analysis; investigation. **Shuang Wang**: Writing—review and editing; validation; supervision. **Jiajia Fang**: Writing—review and editing; funding acquisition; methodology; supervision; resources; project administration.

## CONFLICT OF INTEREST STATEMENT

The authors declare that there are no conflicts of interest regarding the publication of this article.

### PEER REVIEW

The peer review history for this article is available at https://publons.com/publon/10.1002/brb3.3400.

## Supporting information

Table S1 Clinicodemographic characteristics of participants treated with VPA.

## Data Availability

The data supporting this study's findings are available from the corresponding author upon reasonable request.
