# Supplementary material for: Pregnancy‐related knowledge in women with epilepsy in childbearing age: A pilot questionnaire survey from China
Source: Brain Behav. 2024 Feb 10;14(2):e3400. doi: 10.1002/brb3.3400 (PMC10858722; doi:10.1002/brb3.3400)
Supplement: Supplementary file 1 — Table S1 Clinicodemographic characteristics of participants treated with VPA. [file BRB3-14-e3400-s001.docx]

**Table S1.**

**Clinicodemographic characteristics of participants treated with VPA**

| No. | Age | Age of onset | Epilepsy duration | Pregnancy history | Seizure Frequency | ASMs |
| --- | --- | --- | --- | --- | --- | --- |
| 1 | 25 | 3 | 22 | No | Seizure free for several years | VPA, TPM, Others |
| 2 | 23 | 14 | 9 | No | Seizure free for several years | OXC, VPA |
| 3 | 21 | 15 | 6 | No | Seizure free for several years | VPA |
| 4 | 20 | 11 | 9 | No | Seizure free for several years | VPA |
| 5 | 36 | 12 | 24 | Yes | Seizure free for several years | VPA, LTG |
| 6 | 33 | 12 | 21 | No | Seizure free for almost one year | VPA, LEV |
| 7 | 32 | 15 | 17 | No | Seizure free for almost one year | VPA, LEV |
| 8 | 25 | 6 | 19 | No | Seizure free for almost one year | VPA, LCM |
| 9 | 22 | 16 | 6 | No | Seizure free for almost one year | VPA |
| 10 | 47 | 46 | 1 | Yes | Seizure free for almost one year | VPA, LTG |
| 11 | 44 | 40 | 4 | Yes | Seizure free for almost one year | VPA |
| 12 | 34 | 29 | 5 | Yes | Seizure free for almost one year | VPA |
| 13 | 25 | 20 | 5 | No | Several times per year | VPA, LTG, LEV |
| 14 | 19 | 9 | 10 | No | Several times per year | CBZ, VPA, LEV |
| 15 | 33 | 32 | 1 | Yes | Several times per year | VPA |
| 16 | 32 | 31 | 1 | Yes | Several times per year | OXC, VPA, LTG |
| 17 | 30 | 23 | 7 | Yes | Several times per year | VPA, OXC, PER |
| 18 | 27 | 5 | 22 | Yes | Several times per year | CBZ, VPA, LEV, PER, others |
| 19 | 25 | 17 | 8 | Yes | Several times per year | VPA, CBZ |
| 20 | 27 | 2 | 25 | No | Several times per month | CBZ, VPA, LEV, PER |
| 21 | 23 | 4 | 19 | No | Several times per month | VPA, LEV, PER |
| 22 | 19 | 12 | 7 | No | Several times per month | VPA, LTG, others |
| 23 | 43 | 42 | 1 | Yes | Several times per month | VPA, LTG |
| 24 | 40 | 13 | 27 | Yes | Several times per month | LTG, LEV, VPA |
| 25 | 36 | 16 | 20 | Yes | Several times per month | VPA, LCM, OXC, LEV |
| 26 | 31 | 18 | 13 | Yes | Several times per month | OXC, VPA, LEV |
| 27 | 25 | 5 | 20 | Yes | Several times per month | LTG, OXC, VPA |

Abbreviations: VPA: Valproic acid; TPM: Topiramate; OXC: Oxcarbazepine; LTG: Lamotrigine; LEV: Levetiracetam; LCM: Lacosamide
